# COVID-19 in otolaryngologist practice: a review of current knowledge

**DOI:** 10.1007/s00405-020-05968-y

**Published:** 2020-04-18

**Authors:** Joanna Krajewska, Wojciech Krajewski, Krzysztof Zub, Tomasz Zatoński

**Affiliations:** 1 Department and Clinic of Otolaryngology, Head and Neck Surgery, Medical University in Wroclaw, Borowska 213 Street, 50556 Wroclaw, Poland; 2 Department and Clinic of Urology and Urological Oncology, Medical University in Wroclaw, Wroclaw, Poland

**Keywords:** COVID-19, SARS-CoV-2, Otolaryngological manifestations, Olfaction, ENT

## Abstract

**Purpose:**

Otorhinolaryngological manifestations are common symptoms of COVID-19. This study provides a brief and precise review of the current knowledge regarding COVID-19, including disease transmission, clinical characteristics, diagnosis, and potential treatment. The article focused on COVID-19-related information useful in otolaryngologist practice.

**Methods:**

The Medline and Web of Science databases were searched without a time limit using terms “COVID-19”, “SARS-CoV-2” in conjunction with “otorhinolaryngological manifestation”, “ENT”, and “olfaction”.

**Results:**

The most common otolaryngological dysfunctions of COVID-19 were cough, sore throat, and dyspnea. Rhinorrhea, nasal congestion and dizziness were also present. COVID-19 could manifest as an isolated sudden hyposmia/anosmia. Upper respiratory tract (URT) symptoms were commonly observed in younger patients and usually appeared initially. They could be present even before the molecular confirmation of SARS-CoV-2. Otolaryngologists are of great risk of becoming infected with SARS-CoV-2 as they cope with URT. ENT surgeons could be easily infected by SARS-CoV-2 during performing surgery in COVID-19 patients.

**Conclusion:**

Ear, nose and throat (ENT) symptoms may precede the development of severe COVID-19. During COVID-19 pandemic, patients with cough, sore throat, dyspnea, hyposmia/anosmia and a history of travel to the region with confirmed COVID-19 patients, should be considered as potential COVID-19 cases. An otolaryngologist should wear FFP3/N95 mask, glasses, disposable and fluid resistant gloves and gown while examining such individuals. Not urgent ENT surgeries should be postponed. Additional studies analyzing why some patients develop ENT symptoms during COVID-19 and others do not are needed. Further research is needed to determine the mechanism leading to anosmia.

## Introduction

At the end of 2019 in Wuhan, a large city in the Hubei Province of China, a novel coronavirus, Severe Acute Respiratory Syndrome Coronavirus 2 (SARS-CoV-2), was considered as the cause of a number of lower respiratory tract infections [1]. On February 11, 2020, the new disease caused by the SARS-CoV-2 virus was officially termed “COVID-19” by WHO [1]. The high potential of human to human transmission led to rapid COVID-19 epidemic in China and subsequent global pandemic [1]. On March 30, 2020, a total of 638.146 confirmed cases of COVID-19 and 30.039 deaths were reported by WHO.

### Aim of the study

The main aim of this study was to provide a brief and precise review of the current knowledge regarding COVID-19, including disease transmission, clinical characteristics, diagnosis and potential treatment. The article focused on information that, in our opinion, could be useful in otolaryngologist practice. We emphasized the role of otolaryngologist in rapid COVID-19 diagnosis. We also implied the high risk of becoming infected with SARS-CoV-2 as a results of the practice of the ENT specialist.

## Methods

The Medline and Web of Science databases were searched without time limit but focusing on the newest report, using the terms “COVID-19”, “SARS-CoV-2”, “novel coronavirus”, and “coronavirus from Wuhan” in conjunction with “otorhinolaryngological manifestation”, “ENT”, “ear”, “nose”, “throat’’, “oral cavity”, “pharynx”, “larynx”, “hearing”, “vertigo”, “head and neck”, and “olfaction”. Boolean operators (NOT, AND, OR) were also used in succession to narrow and broaden the search. Auto alerts in Medline were also considered, and the reference lists of original articles and review articles were searched for further eligible sources. Opinions of medical societies were also included if applicable. The search included articles without language limitations.

A total of 1790 articles were originally identified using our search criteria. 1740 articles were excluded after abstract or full-text analysis because they did not exactly address the topic. Therefore, the total number of 50 studies were finally chosen to prepare this manuscript. Studies on which this article was prepared were not limited to large cohorts, as a vast majority of reports were based on small cohorts. Because of COVID-19 novelty, randomized controlled studies and precise recommendations for COVID-19 management are not available yet. We presume that even reports based on sparse cohorts could be valuable at this time and may lead to further better disease understanding and treatment.

### Origin, transmission, and characteristics of SARS-CoV-2

Human SARS-CoV-2 express 96.2% genomic similarity to bats’ coronavirus. Because of this high genomic similarity bats have been considered as a natural virus host. Therefore, it was speculated that human SARS-CoV-2 might be transmitted to humans from bats through other mammalian hosts [1].

According to epidemiologic studies, the first human SARS-CoV-2 infection presumably took place in Wuhan’s seafood market where live animals were sold [1]. First COVID-19 cases were confirmed in individuals who had previously visited this market [1]. Nevertheless, the following COVID-19 cases were not exposed to this kind of seafood market- related infection. Therefore, a potential human to human transmission was suggested to be the main source of the virus spread [1]. The virus is transmitted between individuals through respiratory droplets that are produced by an infected person while sneezing, coughing or talking and staying in the short distance from another person. Direct contact with a person with COVID-19 or a direct contact with the surfaces contaminated with SARS-CoV-2 with subsequent contact with own nasal cavity, oral cavity or eyes are also sources of infection [1]. Oro-fecal transmission of the virus is presumably also possible. It was suggested that the two meters distance between an infected and non-infected person should be enough to avoid infection [2]. Van Doremalen et al. conducted an experimental study in which they reported that SARS-CoV-2 was able to remain viable in aerosols for 3 h [3]. SARS-CoV-2 survived up to 72 h on plastic and stainless steel, on copper the virus was not detected after 4 h, while on cardboard no viable virus was found after 24 h [3].

The incubation time for COVID-19 since the exposure to SARS-CoV-2 is believed to reach 14 days, nevertheless, the majority of patients develop COVID-19 disease after 4–5 days (range between 2 and 7 days) after being infected [4, 5]. COVID-19 remains contagious even during the latency period, thus patients before clinical COVID-19 presentation can transfer the virus to others [1].

It was reported that the median time to no detectable SARS-CoV-2 RNA in oropharyngeal samples in patients with COVID-19 reached 20 days (range between 8 and 37 days) [6]. Samples obtained from patients with a mild or moderate form of the disease more quickly became negative for SARS-CoV-2 than samples obtained from severe COVID-19 cases [6].

### Clinical manifestation of COVID-19 – important knowledge for otolaryngologists

Fever, fatigue and dry cough are considered to be the most common manifestations of COVID-19 [7–9]. Anorexia, dyspnea, sputum production, and myalgias are reported in more than 25% of cases [7]. Sore throat, rhinorrhea, headaches, nausea, and diarrhea are less frequent and are mainly observed in mild or moderate forms of the disease [7]. Cough, dyspnea, sore throat, rhinorrhea, nasal congestion, throat congestion, tonsils edema, enlarged cervical lymph nodes or dizziness are symptoms that otolaryngologist could encounter while examining patients with COVID-19.

It was recently reported that COVID-19 led to hyposmia/anosmia and taste disturbances [10]. South Korea, China and Italy presented that a significant number of individuals with COVID-19 was affected by hyposmia/anosmia. A few cases were also detected in Germany, while in South Korea 30% of infected individuals developed hyposmia/anosmia. There are also reports implying that COVID-19 may present as isolated anosmia [10]. Researches from various countries observed patients with COVID-19 presenting isolated anosmia, without any other symptoms [10]. They suggested that these individuals could be the hidden carries of SARS-CoV-2 as they do not meet the current criteria for diagnosing COVID-19. These patients could be the source of the rapid spread of COVID-19.

Professor Hopkins and Kumar from the Rhinological Society recommended that oral corticosteroids should not be incorporated in the treatment of the new-onset anosmia during the COVID-19 pandemic, as they may exacerbate the severity of COVID-19 disease [10]. Nasal steroids are also not recommended for the sudden loss of smell [11]. According to the available reports, patients below 40 years of age are mostly predisposed to develop the form of COVID-19 that is only manifested by hyposmia/anosmia or taste disturbances [10, 11]. Patients with sudden anosmia should be tested for SARS-Cov-2 presence and considered as potential individuals with COVID-19 [11].

The prevalence of particular COVID-19 ear, nose and throat (ENT) manifestations in various reports was presented in Table 1 [4, 7–9, 12–27]. Additional, crucial information found in these studies was also presented in Table 1.

**Table 1:** ENT symptoms of COVID-19 reported in observational studies

Study	Number of studied patients	Population	ENT symptoms	Additional information
Team C-NIRS [12]	295	Australian	– Cough in 54% – Sore throat in 46% – Runny nose in 40% – Dyspnea in 35%	– Criteria for confirmed COVID-19 case: A patient who tested positive to a validated specific SARS-CoV-2 nucleic Acid examination or has the Virus identified by electron microscopy or viral culture – A suspected case should fulfil following criteria: (1) Epidemiological criteria – International travel in the 14 days before COVID-19 onset or – Close contact with a patient with COVID-19 within 14 days before disease development (2) Clinical criteria: – Fever or – Acute respiratory infection (dyspnea, cough, sore throat) with or without fever
Guan et al. [4]	1099	Chinese	– Cough in 67.8% – Dyspnea in 18.7% – Sore throat in 13.9% – Nasal congestion in 4.8% – Throat congestion in 1.7% – Tonsil edema in 2.1% – Enlargement of lymph nodes in 0.2%	– Cough and dyspnea were more commonly observed in patients with severe disease, while nasal congestion and sore throat in individuals with non-severe form of COVID-19
Zhang et al. [15]	140	Chinese	– Cough 75% – Dyspnea in 36.7%	– Allergic diseases and smoking history may potentailly not predispose to COVID-19 – Eosinopenia along with lymphopenia could be a useful tool in diagnosing COVID-19 in individuals with typical clinical symptoms and CT chest abnormalities
Wang et al. [23]	138	Chinese	– Dry cough in 59.4%	– The mean time from Disease onset to dyspnea was 5 days; 7 days to hospital admission, 8 days to ARDS development – Dry cough was a common initial symptom
Liu et al. [8]	137	Chinese	– Cough in 48.2%	– Middle-aged and elderly patients with coexisting chronic diseases were susceptible to respiratory failure
Zhu et al. [19]	116	Chinese	– Cough in 66%	– The majority of patients presented mild form of the disease
Zhao et al. [17]	101	Chinese	– Cough in 62% – Sore throat in 12%	– 70.2% of patients were 21–50 years old – Majority of patients with COVID-19 had typical chest CT abnormalities (GGO, mixed GGO and consolidation, Vascular enhancement in the lesion, traction bronchiectas)
Chen et al. [18]	99	Chinese	– Cough in 82% – Dyspnea in 31% – Sore throat in 5% – Rhinorrhea in 4%	– The majority of patients were men – It was reported that the lower susceptibility of women to develop viral infections could result from the protection of X chromosome and sex hormones, which have a significant role in innate and adaptive immunity
Xu et al. [14]	90	Chinese	– Cough in 63% – Sore throat in 26%	– Chest CT could detect minor pulmonary abnormalities in patients at an early stage of COVID-19 – Initial presentation of bilateral, multifocal, and peripheral ground-glass opacities detected in chest CT might strongly suggest COVID-19
Yang et al. [21]	85	Chinese	– Cough in 58.4% – Dyspnea in 1.3%	– 10.06% of patients had no contact with Hubei Province
Huang et al. [20]	84	Chinese	– Cough in 50%	– Patients with atypical or mild symptoms may not present pulmonary changes during disease appearance. Development of pulmonary infiltrates in CT scan might be delayed and it does not suggest that pneumonia will not develop later
Wu et al. [26]	80	Chinese	– Cough in 63.75% – Dyspnea in 37.5%	– 35% of patients presented a mild form of COVID-19; 61.25% had moderate form; 3.75% of patients suffered from the severe type; nobody was critically ill – 51.25% of patients were diagnosed after the positive result in the first test; 37.5% were tested positive in the second test; 11.25% remained negative until a third test
Xu et al. [9]	62	Chinese	– Cough in 81%	– The median time from exposure to SARS-CoV-2 to the onset of COVID-19 reached 4 days (range: 3–5 days)
Song et al. [24]	51	Chinese	– Cough in 47% – Dizziness in 16%	– All patients except one reported a history of Wuhan contact
Xu et al. [22]	50	Chinese	– Cough in 40% – Sore throat in 8% – Dyspnea in 8%	– Patients with mild form of the disease were significantly younger (mean age 29 years) than those with moderate or severe form
Huang et al. [7]	41	Chinese	– Cough in 76% – Dyspnea in 55%	– Patients requiring hospitalization in an intensive care unit expressed higher plasma levels of IL-2, IL-7, IL-10, GSCF, IP10, MCP1, MIP1A, and TNFα – Majority of patients were men (73%) – 32% had underlying diseases: 20% had diabetes, 15% had hypertension, 15% had cardiovascular disease – 66% had direct exposure to Huanan seafood market
Covid-19 National Emergency Response Center [13]	28	Korean	– Sore throat in 32.1% – Cough in 17.9%	– Secondary COVID-19 infection developed in patients from close contact with an infected individual after staying together for a considerable amount of time
Chang et al. [25]	13	Chinese	– Cough in 46.2% – Nasal congestion in 7.7%	– Majority of the patients with COVID-19 were healthy adults; 1 patient was older than 50 years; 1 was younger than 5 years
Spiteri et al. [27]	9	World Health Organization European Region (excluding United Kingdom)	– Cough in 45% – Sore throat in 6.4% – Rhinorrhea in 6.4% – Dyspnea in 6.4%	– Two cases were asymptomatic and remained so until became SARS-CoV-2 negative – Median hospitalization time was 13 days (range: 8–23 days)
Han et al. [16]	1	Chinese	Patient presented cough with white discharge, stuffy and runny nose and vertigo altogether	– Chest CT imaging accompanied by the detection of SARS-CoV-2 RNA is helpful for the COVID-19 diagnosis – Methylprednisolone in combination with interferon therapy did not significantly improve patient’s condition. LPV-RTV incorporation led to quick improvement of the clinical symptoms

*CT* computed tomography, *GGO* ground glass opacities, *IL-2* interleukin 2, *IL-7* interleukin 7, *IL-10* interleukin 10, *GSCF* granulocyte-colony stimulating factor, *IP10* interferon gamma-induced protein 10, *MCP1* monocyte chemoattractant protein 1, *MIP1A* macrophage Inflammatory protein 1alpha, and *TNFα* tumor necrosis factor alpha; ARDS – acute respiratory distress syndrome

### COVID-19-related crucial information for otolaryngologists/head and neck surgeons

Otolaryngologists, especially ENT surgeons are at very high risk of SARS-Cov-2 infection as they cope with the upper respiratory tract (URT) which is the main reservoir of SARS-CoV-2. According to current recommendations of European Rhinologic Society, all non-urgent ENT surgeries should be postponed because of COVID-19 pandemic [11]. For patients requiring urgent surgery or ENT consult, otolaryngologist should wear fluid-resistant FFP3/N95 mask, disposable and fluid resistant gloves and gown, glasses or full face shield. Double-gloving during operation is recommended for surgeons [2]. A number of staff attending the OR during urgent ENT surgery should be limited to minimum [2].

Tracheostomy is one of the most frequent urgent ENT surgery. Currently, during COVID-19 pandemic, every patient requiring emergency tracheostomy should be considered as a COVID-19 positive as delaying the surgery while waiting for SARS-CoV-2 detection test may lead to patient’s death [28].

For patients with intermittent dyspnea that is potentially reversible, intubation rather than tracheostomy should be performed [28]. High flow oxygen/AIRVO should not be used in these cases [28]. For individuals with constant dyspnea, in whom irreversible cause of dyspnea is strongly suspected, tracheostomy is required [28]. In patients positive for COVID-19 or in those with unknown COVID-19 status, cuffed and non-fenestrated tracheostomy tube should be used to prevent SARS-Cov-2 from aerosolizing [28]. The cuff of the endotracheal tube should not be perforated during the procedure. The mechanic ventilation should be suspended while making the opening in the trachea and during the tracheostomy tube insertion into the trachea [28]. Heat and moisture exchanger (HME) ought to be immediately connected with the tracheostomy to reduce the spread of the virus. Subsequently it should not be disconnected [28]. It is recommended to avoid humidified closed circuits to minimize the chance of the virus-induced space contamination in case of the system disjunction [28]. The closed gear used in patients after tracheostomy should be the same as used for patients connected to a mechanical ventilator [29]. After the surgery, the tracheostomy tube should not be changed until patient’s COVID-19 status is positive or unknown [28]. Suction other than closed in line suction must be avoided while performing respiratory tract toilet [28].

Flexible laryngoscopy is another aerosol-generating procedure that exposes otolaryngologists to COVID-19 infection [29]. It should be performed only if absolutely necessary.

It is recommended that every patient with unknown status of COVID-19 should be examined by otolaryngologist that is fully equipped [29]. It is of great importance as the mean incubation time reaches 5.2 days, with 95% of the distribution at 12.5 days [5]. The minimal personal protective equipment (PPE) includes FF3/N95 mask, gloves, gown, eye protection and a cap [29]. If possible, patients not requiring urgent ENT consult, especially those treated for chronic ENT diseases, should be consulted by phone. Individuals requiring ENT visit are obligated to have their body temperature measured before entering the outpatient clinic [29]. Additionally, their recent travel status should be assessed as the patients could be asymptomatic during the first several days after SARS-CoV-2 infection [29].

Currently, there is no evidence against topical corticosteroids use in patients with chronic nasal corticosteroids use for rhinosinusitis or allergic rhinitis [11].

### COVID-19 diagnosis

Symptoms including fever, unproductive cough and dyspnea in combination with a history of travel to areas with confirmed COVID-19 cases strongly suggest COVID-19 disease [30]. Currently, patients with severe lower respiratory tract infection should be considered as potential SARS-CoV-2 carriers [30]. Nevertheless, to confirm the diagnosis of COVID-19 molecular test must detect SARS-CoV-2 presence [30]. The specific test for SARS-CoV-2 detection is the real-time reverse transcriptase-polymerase chain reaction (RT-PCR) test. Presently, it is the recommended test to diagnose SARS-Cov-2 infection. A positive RT-PCT test for SARS-CoV-2 confirms the diagnosis of COVID-19 in the vast majority of cases, nevertheless, false-positive results can also occur [30]. RT-PCR is considered as highly specific, however, in a number of cases its sensitivity seemed not to be enough to diagnose the disease. RT-PCR sensitivity range differed between reports from various counties. It could be as low as 60–70% [31] or as high as 97% [32]. Keeping in mind such detection discrepancies, doctors should repeat the test after several days.

Molecular examination is performed on specimens obtained mainly from the respiratory tract and sometimes from stool [30]. In severe form of the disease, blood is tested for SARS-CoV-2 presence [30]. Nasopharyngeal swabs, oropharyngeal swabs, bronchoalveolar lavage, endotracheal aspirates or sputum could be taken for testing [30].

Nasopharyngeal swabs are most commonly taken for SARS-CoV-2 examination.

Nasopharyngeal and oropharyngeal swabs are currently recommended for detecting SARS-CoV-2 and diagnosing COVID-19, nevertheless obtaining specimens from this sites may be harmful and may lead to bleeding that is especially important in cases where recurrent analysis is required [33]. Additionally, it was also reported that swabs taken from URT, especially from nasopharynx or oropharynx might not be enough sensitive to detect SARS-CoV-2 [34].

According to current recommendations, two negative tests conducted in at least 24-h interval could exclude COVID-19. Nevertheless, Wu et al. presented a case of COVID-19 with double negative SARS-CoV-2 tests obtained from nasopharyngeal swabs [35]. The authors suggested that in patients with clinical symptoms strongly suggesting COVID-19 disease, sputum or bronchoalveolar lavage fluid (BALF) should be taken for examination [35]. The patient presented in this case was co-infected by influenza A virus that was detected in the nasopharyngeal swab. Simultaneously, the patient was negative for SARS-CoV-2 [35]. This report highlights the possibility of false-negative results for samples obtained from URT while diagnosing COVID-19 [35].

Sputum could be analyzed for SARS-CoV-19 presence however it may be difficult to obtain in non-productive patients [33]. It was speculated that saliva may serve as a potential, non-invasive material for diagnosing COVID-19 [33]. Saliva could be self-collected by a patient by spitting into a sterile container. That could eliminate the exposure of healthcare service to close contact with a patient while taking naso- or oropharyngeal swabs [33]. It was also reported that in several cases saliva was more accurate material to detect coronavirus than nasopharyngeal swab [34]. Authors from China reported that SARS-CoV-2 was detected in saliva specimens obtained from 91.7% of patients with COVID-19 [33]. Saliva was taken into a sterile container from patients while spitting saliva from throat and subsequently analyzed using nucleic acid extraction and RT-PCR test [33].

According to studies, computed tomography (CT) of the chest seemed to be very useful in diagnosing COVID-19. It was suggested that chest CT could be even more sensitive in detecting COVID-19 than repeated RT-PCR test. Ai et al. conducted a large cohort study performed on patients with positive RT-PCR test revealing that the sensitivity of chest CT in implying the presence of COVID-19 reached 97% [32]. The sensitivity of RT-PCR tests and chest CT for diagnosing COVID-19 in suspected individuals reached 59% and 88%, respectively [32]. 60% to 93% of patients in this cohort presented initial positive chest CT suggesting COVID-19 before the initial RT-PCR test detected SARS-CoV-2 [32]. 42% of patients with COVID-19 presented improvement in the follow-up chest CT before the test based on RT-PCR results turned negative [32]. The authors suggested that chest CT could be considered as a sensitive and useful test in detecting COVID-19 in the areas affected by COVID-19 epidemic [32]. CT of the thoracic cavity revealing ground-glass opacities, infiltrates and bronchovascular thickening consolidations strongly suggest SARS-CoV-2 infection [23].

According to previous reports, we speculate that during COVID-19 pandemic, chest CT should be performed in patients before ENT operations. It could be of great value in individuals with negative RT-PCR.

There are currently no laboratory abnormalities specific for COVID-19 diagnosis. According to various authors, complete blood count usually revealed the normal or decreased level of white blood cells and thrombocytes, and reduced number of lymphocytes [30]. The levels of erythrocyte sedimentation rate and C-reactive protein were mainly increased, while procalcitonin remained normal in the majority of cases [30]. Increased levels of D-dimer, serum creatinine, creatinine phosphokinase, lactate dehydrogenase, prothrombin time, and aminotransferases namely alanine transaminase and aspartate transaminase, usually indicated severe form of COVID-19 [30]. High D-dimer concentration and significant lymphopenia were correlated with higher mortality [30]. Patients with ENT manifestations of COVID-19 may present similar laboratory abnormalities to individuals in alike disease stage but with other COVID-19 symptoms.

It was implied that the loop-mediated isothermal amplification (LAMP) assay could be a potentially useful tool in diagnosing COVID-19 because of its diagnostic sensitivity exceeding 95% [36]. LAMP reaction is a novel nucleic acid amplification analysis that amplifies DNA [36]. It is characterized by very specific, efficient and quick test [36]. LAMP technology is believed to be of higher stability and sensitivity than PCR [36].

High levels of interleukin-1B (IL-1B), interferon- γ (IFN-γ), interferon gamma-induced protein 10 (IP10), and monocyte chemoattractant protein 1 (MCP1) were found in patients with COVID-19. T-helper-1 (Th1) cell response was potentially dominant in infected individuals [7]. Nevertheless, the levels of IL-4 and IL-10 that are related to T-helper-2 (Th2) cell response, were also elevated [7]. Additionally, patients with severe form of the disease, requiring hospitalization in the intensive care unit (ICU), expressed high levels of granulocyte-colony stimulating factor (GCSF), IP10, MCP1, macrophage Inflammatory protein 1alpha (MIP1A), and tumor necrosis factor alpha (TNFα) [7]. Concentrations of these molecules were significantly higher than in patients with less severe disease [7]. The authors speculated that these molecules may potentially reflect a severe form of COVID-19 [7].

### Vaccine

Vaccine against SARS-CoV-2 is not available yet. The most promising target in developing a vaccine against SARS-CoV-2 seems to be the viral spike protein (S protein) [37]. The first vaccine is examined in the clinical trial (Phase 1) in human beings in the United States [37]. It uses a messenger RNA platform to achieve S protein expression to stimulate an immune response [37].

### Therapy for COVID-19 (Fig. 1)

Previously known SARS-CoV, and novel SARS-CoV-2 express genomic similarity (approximately 82% similarity), thus therapeutic option used for SARS-CoV could potentially be useful in treating SARS-CoV-2 infection [1, 36]. Partial genomic similarity was also observed between SARS-CoV-2 and Middle East Respiratory Syndrome coronavirus (MERS-CoV).

**Fig. 1:**
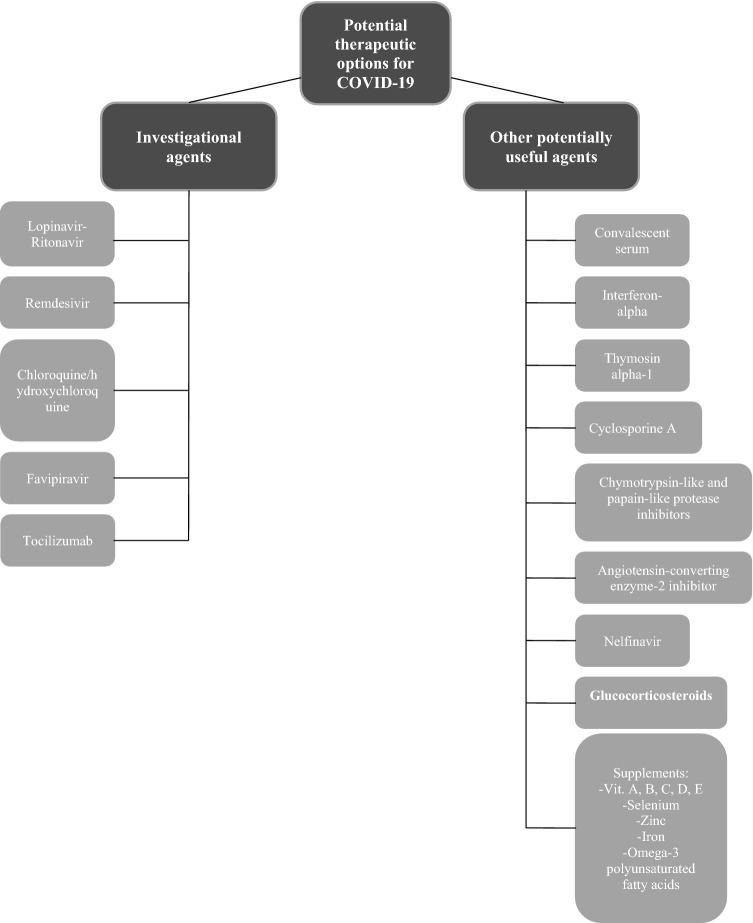
Potential therapeutic options for COVID-19

No therapeutic agent has already been proven to be efficient in SARS-Cov-2 infection treatment. No drug is currently approved for COVD-19. However, several agents are currently investigated in clinical trials [1].

### Agents being investigated in COVID-19

There are currently several agents used in COVID-19 therapy. Randomized controlled studies analyzing their potential positive effects in COVID-19 management are lacking. Reports on these agents’ use in COVID-19 are mainly based on in vitro or extrapolated evidence. Clinical usefulness of these drugs appeared in case reports.

#### Lopinavir (LPV)-Ritonavir (RTV)

Therapy based on combined LPV and RTV, protease inhibitors recommended for HIV-1 treatment, showed antiviral activity against SARS-CoV in in vitro study [38]. Promising effects were also found in managing MERS-CoV with LPV-RTV in a study on animals [39].

The efficiency of LPV-RTV use for COVID-19 treatment has not already been established. Further clinical studies are required to assess the potential benefits of these agents in COVID-19.

Cao et al. conducted a study on 199 patients with COVID-19 [40]. The authors reported that LPV-RTV therapy was not effective in adults with severe COVID-19. The use of LPV-PTV (dosage 400 and 100 mg, respectively) two times a day for two weeks showed no significant difference in the time to clinical improvement, duration of the stay in ICU, duration of mechanical ventilation, duration of oxygen support, or in mortality rate from patients undergoing standard care without LPV-RTV under the observation for 28 days [40].

Deng et al. found that therapy based on LPV-RTV in combination with Arbidol, a drug used against influenza virus, was more efficient than LPV-RTV use only in confirmed COVID-19 cases [41].

#### Remdesivir

Remdesivir, a novel nucleotide analogue could be a potential therapeutic option for COVID-19 [42]. It appeared to have activity against SARS-CoV-2 in in vitro study [42]. Remdesivir was clinically used for the first time in 35-years old American with COVID-19 when the patient’s clinical status started to exacerbate [43]. Currently, there are two ongoing large cohort clinical trials designed to analyze the efficiency and safety of remdesivir in patients with mild, moderate, and severe COVID-19 [44]. Preliminary results of these studies have not been reported yet.

#### Chloroquine/hydroxychloroquine

Chloroquine/hydroxychloroquine expressed promising results in COVID-19 treatment in various studies [45, 46]. Chloroquine and hydroxychloroquine presented antiviral activity against SARS-CoV-2 in in vitro and in vivo studies [46, 47]. Stronger antiviral function was observed for hydroxychloroquine than for chloroquine [46]. Chloroquine was able to decrease the length of hospitalization and to improve COVID-19 pneumonia therapy. It expressed positive results with potential safety in fighting COVID-19-associated pneumonia in the Chinese multicenter clinical trials [48]. In a study conducted by Gautret et al., 100% of individuals with COVID-19 undergoing therapy based on hydroxychloroquine and azithromycin were healed, while only 57.1% of patients on hydroxychloroquine alone and only 12.5% of individuals from control group improved after the therapy [45]. Chloroquine is currently recommended to be incorporated in the upcoming version of the “Guidelines for the Prevention, Diagnosis, and Treatment of Pneumonia Caused by COVID-19” issued by the National Health Commission of the People's Republic of China [48].

#### Favipiravir

Favipiravir is a novel RNA-dependent RNA polymerase inhibitor [49]. It was approved for novel influenza virus in China [49]. Favipiravir expresses its inhibiting activity against viral RNA polymerase after initially being transformed to an active phosphoribosylated form (favipiravir-RTP) in cells, where it is identified as a substrate by the viral RNA polymerase [49]. It could potentially be useful in COVID-19 treatment as SARS-CoV-2 is a RNA virus [49]. Encouraging results were already achieved by a clinical trial conducted in the Clinical Medical Research Center of the National Infectious Diseases and the Third People's Hospital of Shenzhen [49]. Authors of this trial reported that favipiravir expressed stronger antiviral activity than LPV-RTV with significantly less adverse effects [49].

#### Tocilizumab

Tocilizumab is a recombinant humanized monoclonal antibody against the interleukin-6.

(IL-6) receptor [44]. It showed promising results in treating severely or critically ill individuals with COVID-19 with coexisting massive changes in lungs and significantly elevated levels of IL-6 [44].

### Other potentially useful agents in COVID-19

#### Convalescent serum

Sera obtained from convalescent patients could potentially be used in COVID-19 therapy in subjects with early symptoms [50]. It could prevent COVID-19 development in exposed individuals [50].

#### Interferon-alpha (INF-alpha)

Zhang et al. reported the potential therapeutic effect of INF-alpha and combined INF-alpha-2a with ribavirin in patients with SARS and severe MERS-CoV, respectively [47].

#### Thymosin alpha-1

Thymosin alpha-1 express the ability to elevate the resistance to glucocorticosteroids-induced death of thymocytes. It was observed that thymosin alpha-1 acted as an immune booster in patients with SARS and was able to restrain the spread of the infection [47]. In patients with COVID-19 in whom incorporating glucocorticosteroids is planned, thymosin alpha-1 should be considered before glucocorticosteroid use to prevent glucocorticosteroid-induced death of thymocytes [47].

#### Cyclosporine A

It was speculated that non-immunosuppressive derivatives of cyclosporine A might act as COVID-19 inhibitors [47].

#### Chymotrypsin-like (3C-like) and papain-like protease (PLP) inhibitors

Therapy targeting coronavirus proteases, namely chymotrypsin-like (3C-like) and papain-like protease (PLP) could potentially be useful in COVID-19 treatment [47]. 3C-like protease was observed to be encoded in COVID-19 [47]. Cinanserin express the ability to suppress 3C-like protease subsequently implying its potential usefulness in facing COVID-19 [47]. Cinanserin was able to suppress SARS-CoV replication [47]. Flavonoids presented the inhibitory effects against MERS-CoV and SARS-CoV chymotrypsin-like proteases [47]. Diarylheptanoid, a PLP inhibitor, expressed the ability to inhibit SARS-CoV PLP [47]. Targeting coronavirus proteases could potentially be useful in facing COVID-19 [47].

#### Angiotensin-converting enzyme-2 (ACE2) inhibitors

ACE2 was found to be a critical receptor of SARS-CoV-2 invasion [1]. SARS-CoV-2 Spike (S) glycoprotein binding with host ACE2 enables the virus to invade the human organism [1]. Suppressing SARS-CoV-2 S glycoprotein from linking with ACE2 could be a promising therapeutic option for SARS-CoV-2 infection development [1].

Recombinant human monoclonal antibody scFv80R against S1 domain of the SARS-CoV was found [47]. It was reported that this antibody was able to suppress SARS-CoV S glycoprotein from binding to ACE2 and counteract SARS-CoV [47].

Emodin expressed antiviral activity [47]. It suppressed SARS-CoV and ACE2 fusion because of its ability to compete with SARS-CoV S glycoprotein [47]. This observation implied the potential usefulness of emodin in COVID-19 therapy [47]. Similar competition with SARS-CoV S glycoprotein for connection to the ACE2 receptor was also observed for promazine [47].

#### Nelfinavir

Nelfinavir, a HIV protease inhibitor, was able to inhibit SARS-CoV, thus it could be also considered as a potential therapeutic option for SARS-CoV-2 [47].

#### Glucocorticosteroids

World Health Organization (WHO) and the Centers for Disease Control and Prevention (CDC) recommended that glucocorticosteroids should not be commonly administered in individuals with COVID-19 for managing SARS-CoV-2-induced pneumonia or acute respiratory distress syndrome (ARDS) unless required for another reason, like worsening of the chronic obstructive pulmonary disease, asthma or septic shock [44]. Nevertheless, it was reported that in patients with COVID-19 pneumonia who progressed to ARDS, methylprednisolone brought favorable results [44].

#### Supplements

It was speculated that Vitamin A, B, C, D, E, omega-3 polyunsaturated fatty acids, selenium, zinc and iron supplementation could be beneficial in COVID-19 management [47].

## Conclusion

Otorhinolaryngological manifestations are not rare symptoms of COVID-19, especially in mild or moderate form of the disease. The most common ENT dysfunctions observed in patients infected with SARS-CoV-2 are cough, mainly dry, sore throat and dyspnea. Rhinorrhea, nasal congestion and dizziness may also be present. COVID-19 could also manifest as a sudden hyposmia or anosmia not accompanied by any other symptom. Whether SARS-CoV-2-induced hyposmia/anosmia is reversible remain unknown. URT symptoms are commonly observed in younger patients and usually appear initially. They may precede the development of severe COVID-19. Mild cases of COVID-19 without clinical pneumonia could represent the clinical presentation of the disease in young, healthy individuals. ENT symptoms may be present before the patient tested positive for SARS-CoV-2 in molecular analysis.

Otolaryngologist is of great risk of becoming infected with SARS-CoV as they cope with URT during performing a consult, clinical examination, sample taking and a surgery. ENT specialist is one of the specialists that patients with COVID-19 contact most commonly. During COVID-19 pandemic, every patient with cough, sore throat, dyspnea, hyposmia/anosmia and a history of travel to the region with confirmed COVID-19 cases, should be considered as a potential COVID-19 case. Otolaryngologist should wear fluid-resistant FFP3/N95 mask, disposable and fluid resistant gloves and gown, glasses or a full face shield when examining such individual. According to previous reports, we speculate that during COVID-19 pandemic, chest CT should be performed in patients before ENT operations. It could be of great value in individuals with negative RT-PCR.

Additional studies analyzing why not all patients develop ENT symptoms during SARS-CoV-2 infection are needed. Further research is needed to determine the mechanism leading to loss of smell.
